# Lack of Correlation between the Kinase Activity of LRRK2 Harboring Kinase-Modifying Mutations and Its Phosphorylation at Ser910, 935, and Ser955

**DOI:** 10.1371/journal.pone.0097988

**Published:** 2014-05-16

**Authors:** Genta Ito, Tetta Fujimoto, Shogo Kamikawaji, Tomoki Kuwahara, Takeshi Iwatsubo

**Affiliations:** 1 Department of Neuropathology, Graduate School of Medicine, The University of Tokyo, Tokyo, Japan; 2 Department of Neuropathology and Neuroscience, Graduate School of Pharmaceutical Sciences, The University of Tokyo, Tokyo, Japan; National Center of Neurology and Psychiatry, Japan

## Abstract

Leucine-rich repeat kinase 2 (LRRK2) is extensively phosphorylated in cells within a region amino-terminal to the leucine-rich repeat domain. Since phosphorylation in this region of LRRK2, including Ser910, Ser935, Ser955, and Ser973, is significantly downregulated upon treatment with inhibitors of LRRK2, it has been hypothesized that signaling pathways downstream of the kinase activity of LRRK2 are involved in regulating the phosphorylation of LRRK2, although the precise mechanism has remained unknown. Here we examined the effects of LRRK2 inhibitors on the phosphorylation state at Ser910, Ser935, and Ser955 in a series of kinase-inactive mutants of LRRK2. We found that the responses of LRRK2 to the inhibitors varied among mutants, in a manner not consistent with the above-mentioned hypothesis. Notably, one of the kinase-inactive mutants, T2035A LRRK2, underwent phosphorylation, as well as the inhibitor-induced dephosphorylation, at Ser910, Ser935, and Ser955, to a similar extent to those observed with wild-type LRRK2. These results suggest that the kinase activity of LRRK2 is not involved in the common mechanism of inhibitor-induced dephosphorylation of LRRK2.

## Introduction

Parkinson’s disease (PD) is one of the most common neurodegenerative disorders pathologically characterized by neuron loss in the substantia nigra accompanied by formation of Lewy bodies [1]–[3]. Most PD patients develop the disease in a sporadic manner, whereas a subset of patients inherits PD as autosomal dominant or recessive traits (familial PD; FPD) [4]. The *LRRK2* gene has been identified as a causative gene for PARK8, an autosomal-dominant form of FPD [5], [6], and six missense mutations (i.e., R1441C/G/H, Y1699C, G2019S, I2020T) have so far been described in PARK8 families [7]. Moreover, SNPs around the *LRRK2* locus have been reported to be associated with the risk for sporadic PD in two independent genome-wide association studies, implicating LRRK2 in the pathogenesis of PARK8 as well as of sporadic PD [8], [9].

**Figure 1:**
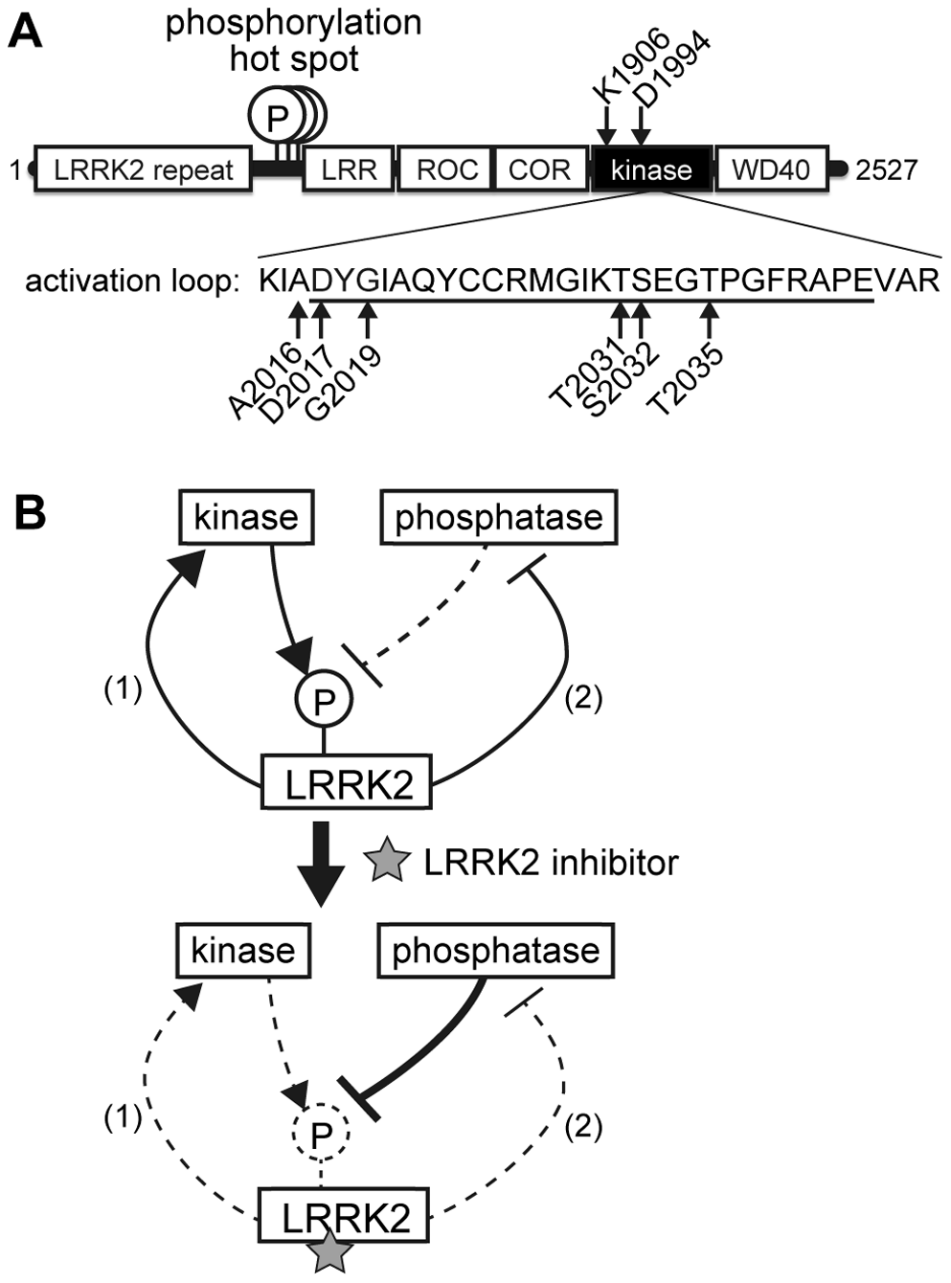
LRRK2 and the hypothetical mechanism of its inhibitor-induced dephosphorylation. (A) Schematic depiction of the domain structure of LRRK2. The phosphorylation hot spot is located amino-terminal to the LRR domain. The residues mutated in this study (Lys1906, Asp1994, Ala2016, Asp2017, Gly2019, Thr2031, Ser2032, and Thr2035) are also indicated. LRR: leucine-rich repeat, ROC: Ras of complex proteins, COR: carboxyl-terminal of ROC. (B) The hypothetical mechanism of the inhibitor-induced dephosphorylation. Dashed lines represent inhibited pathways.

It has been repeatedly shown that LRRK2 is phosphorylated in cells at multiple sites amino-terminal to the leucine-rich repeat (LRR) domain [10], [11]. These sites including Ser910, Ser935, Ser955, and Ser973 have been identified as those intracellularly phosphorylated by mass spectrometric analyses (Fig. 1A; phosphorylation hot spot) [12]–[14]. Since LRRK2 does not phosphorylate itself at these sites *in vitro*, it has been believed that these phosphorylations do not represent autophosphorylation but are executed by other kinases [15], [16]. Indeed, Dzamko and colleagues have reported that, in macrophages, IκB kinases phosphorylate LRRK2 at Ser935 [17], although responsible kinases in non-immune cells remain unidentified. Phosphorylation at Ser910 and Ser935 of LRRK2 has been reported to be required for binding of 14-3-3 proteins, although the physiological significance of this interaction is yet to be elucidated [13], [18]. Importantly, it has been reported that LRRK2 harboring familial Parkinson mutations exhibits reduced cellular phosphorylation by an unknown mechanism, implicating the disturbed cellular phosphorylation in the toxic mechanism of familial Parkinson mutant LRRK2 [16].

Interestingly, it has been shown that phosphorylation of LRRK2 at these sites are rapidly downregulated upon treatment of Swiss 3T3 cells with inhibitors of LRRK2 [15]. This observation has been confirmed in other cell lines as well as in mice using various types of LRRK2 inhibitors [16], [19]–[27]. These data led to the hypothesis that the kinase or phosphatase responsible for phosphorylation of LRRK2 is regulated by the kinase activity of LRRK2 (Fig. 1B). Given that a physiological substrate of LRRK2 is yet to be identified, this ‘inhibitor-induced dephosphorylation’ is currently considered as an alternative readout of the intracellular inhibition of the kinase activity of LRRK2 [28].

However, LRRK2 harboring mutations either inactivating (e.g. K1906M) or increasing (e.g. G2019S) its kinase activity is phosphorylated at these sites at similar levels to those in wild-type (WT) LRRK2 [16], [29]. Assuming that the kinase activity of LRRK2 regulates the phosphorylation of LRRK2 in cells, kinase-inactive and hyperactive LRRK2 should have decreased and increased phosphorylation, respectively. Thus, the above-mentioned hypothesis seems to be inconsistent in terms of the correlation between the kinase activity and the phosphorylation of LRRK2. To clarify whether inhibition of the kinase activity of LRRK2 is involved in the mechanism of inhibitor-induced dephosphorylation of LRRK2 within cells, we compared the response to three ATP-competitive LRRK2 inhibitors of seven kinase-inactive, two hyperactive, and one inhibitor-resistant mutants of LRRK2. Unexpectedly, we found that the basal phosphorylation status as well as the response of LRRK2 to the inhibitors strikingly varied among mutants, and that one kinase-inactive mutant (i.e. T2035A) underwent the basal phosphorylation as well as the inhibitor-induced dephosphorylation in a similar manner to WT LRRK2. These results provide strong negative evidence against the uniform hypothesis that the phosphorylation of LRRK2 is regulated by a signaling mechanism downstream of the kinase activity of LRRK2.

## Materials and Methods

### Construction of Expression Plasmids

The expression plasmids encoding full-length human LRRK2 (wild-type, K1906A, K1906M, D1994A, D1994N, A2016T, D2017A, G2019S, T2031S, S2032A, and T2035A) cloned in the p3×FLAG-CMV-10 vector (Sigma) were constructed as described previously [30]–[32]. Following oligonucleotides were used as forward primers: 5′-gaagtggctgtggcgatttttaataaac-3′ for K1906A, 5′-gattatataccgaaacctgaaaccc-3′ for D1994N, 5′-catcattgcaaagattactgactacggcattg-3′ for A2016T, 5′-caaagattgctgcctacggcattg-3′ for D2017A, 5′-ggataaaatcatcagagggcac-3′ for T2031S, 5′-ggataaaaacagcagagggcac-3′ for S2032A, and the corresponding complementary sequences were used as reverse primers. Primers for other mutants were described previously [30]–[32].

### Cell Culture, Transfection and Treatment with Inhibitors

HEK (human embryonic kidney) 293 cells were maintained in DMEM (Dulbecco’s modified Eagle’s medium) supplemented with 10% (v/v) fetal bovine serum and 100 units/ml penicillin/100 µg/ml streptomycin at 37°C in 5% CO_2_ atmosphere. Transient expression in HEK293 cells was performed by transfecting the plasmids using FuGENE6 (Roche) or polyethylenimine (Sigma) according to the manufacturer’s instructions. LRRK2-IN-1 was provided by Professor Dario Alessi (University of Dundee). Sunitinib and H-1152 were purchased from Sigma and Calbiochem, respectively. LRRK2-IN-1 and sunitinib were dissolved in dimethyl sulfoxide (DMSO). H-1152 was dissolved in sterilized distilled water (SDW). Thirty-six hours after transfection, cells were treated with inhibitors or an equivalent volume of solvents (DMSO or SDW). The final concentrations of solvents were 0.1% (v/v) for DMSO and 1% (v/v) for SDW.

### Antibodies and Immunochemical Analysis

Rabbit monoclonal antibodies for human LRRK2 (MJFF2; #3514-1), for the phosphorylated form of LRRK2 (anti-pSer910 LRRK2 (#5098-1); anti-pSer935 LRRK2 (#5099-1)), and autophosphorylated form of LRRK2 (anti-pThr1410 LRRK2 (#7125-1); anti-pThr1491 LRRK2 (#7058-1)) were purchased from Epitomics. A rabbit monoclonal antibody recognizing anti-pSer955 LRRK2 was purchased from Abcam (#ab169521). A rabbit monoclonal antibody recognizing phosphorylated LRRKtide was purchased from Cell Signaling Technology (#3726). A rabbit polyclonal antibody recognizing phosphorylated Thr1357 of LRRK2 was generated as described previously [33].

### Quantitative Analysis of the Kinase Activity of LRRK2

An amino-terminally biotin-tagged LRRKtide (bLRRKtide; biotin-RLGRDKYKTLRQIRQ) (BEX, Japan) was used as a substrate. Transfected HEK293 cells in a 10 cm dish were lysed in 1 ml of the lysis buffer [50 mM Tris-HCl (pH 7.6), 150 mM NaCl, 0.5% (v/v) NP-40, Complete protease inhibitor cocktail (Roche), and PhosSTOP phosphatase inhibitor cocktail (Roche)] for 30 minutes at 4°C. Cleared lysates were incubated with 10 µl of 50% (v/v) M2-agarose (Sigma) for 1 h at 4°C. The beads were washed three times with the wash buffer 1 [50 mM Tris-HCl (pH 7.6), 150 mM NaCl, 0.5% (v/v) NP-40] and then twice with the wash buffer 2 [50 mM Tris-HCl (pH 7.6), 150 mM NaCl, 0.02% (v/v) Tween-20]. The beads were incubated for 15 minutes in 10 µl of the reaction buffer [20 mM Tris-HCl (pH 7.5), 20 mM NaCl, 2 mM DTT, 0.02% (v/v) Tween-20, 100 µM ATP, Complete protease inhibitor cocktail EDTA-free (Roche), and PhosSTOP phosphatase inhibitor cocktail (Roche)] containing 300 µM bLRRKtide. The reactions were stopped by addition of 990 µl of the ice-cold stop buffer [50 mM Tris-HCl (pH 7.6), 150 mM NaCl, 0.5% (v/v) NP-40, and 20 mM EDTA], and the beads were removed by passing through empty spin columns. Fifty microliter of the diluted reaction mixtures were applied onto a 96-well plate coated with streptavidin (Thermo Fisher Scientific) and incubated for 1 h at 37°C. The plates were washed four times with DPBS-T [2.68 mM KCl, 1.47 mM KH_2_PO_4_, 137 mM NaCl, 8.1 mM Na_2_HPO_4_, 0.05% (v/v) Tween-20 (pH 7.4)]. Phosphorylation-specific antibodies (anti-phospho-LRRKtide) diluted in the buffer C [20 mM phosphate buffer (pH 7.0), 0.5 M NaCl, 2 mM EDTA, 10% (w/v) Block Ace (DS Pharma Biomedical, Japan), 0.2% (w/v) BSA] were added to the plates followed by incubation at 4°C overnight. The plates were washed four times with DPBS-T, and secondary antibodies conjugated with horseradish peroxidase (anti-rabbit IgG from donkey; GE Healthcare) diluted in the buffer C were added to the plates followed by incubation for 1 h at room temperature. The plates were washed eight times with DPBS-T and developed using TMB Microwell Peroxidase Substrate System (KPL). For detecting *in vitro* autophosphorylation of LRRK2, immunoprecipitated LRRK2 was incubated in 20 µl of the reaction buffer, and the reaction was stopped by addition of 20 µl of 2×SDS-PAGE sample buffer and boiling. Samples were analyzed by immunoblotting using antibodies recognizing autophosphorylation sites of LRRK2.

### Statistic Testing

Prior to examining the statistical significance of differences, a normal distribution of the data was examined by Shapiro-Wilk test using IBM SPSS Statistics Version 21. The statistical significance of differences between data following a normal distribution was examined by the Student’s t-test or one-way/two-way ANOVA followed by Bonferroni test as indicated in the figure legend. The statistical significance of differences between data not following a normal distribution was examined by the Kruskal-Wallis test if possible. Otherwise Student’s t-test or ANOVA followed by Bonferroni test was carried out and asterisks showing the statistical significance were marked with parentheses. Statistical tests were done using Prism 6 (GraphPad), and differences were considered to be statistically significant when p<0.05.

## Results and Discussion

Given that there exists equilibrium between phosphorylation and dephosphorylation of proteins within cells, the currently prevailing understanding of the molecular mechanism underlying the inhibitor-induced dephosphorylation of LRRK2 is based on an assumption that the equilibrium is disrupted by inhibition of the kinase activity of LRRK2 (Fig. 1B). Based on this assumption, two scenarios in the regulation of the phosphorylation of LRRK2 are conceivable: (1) a kinase which phosphorylates LRRK2 within the hot spot including Ser910, Ser935, and Ser955 is activated by the kinase activity of LRRK2, or (2) a phosphatase which dephosphorylates these residues of LRRK2 is inhibited by the kinase activity of LRRK2. In either case, inhibition of the kinase activity of LRRK2 by inhibitors disrupts the equilibrium of the phosphorylation in the hot spot, which results in rapid dephosphorylation.

### Phosphorylation and Inhibitor-induced Dephosphorylation of LRRK2

**Figure 2:**
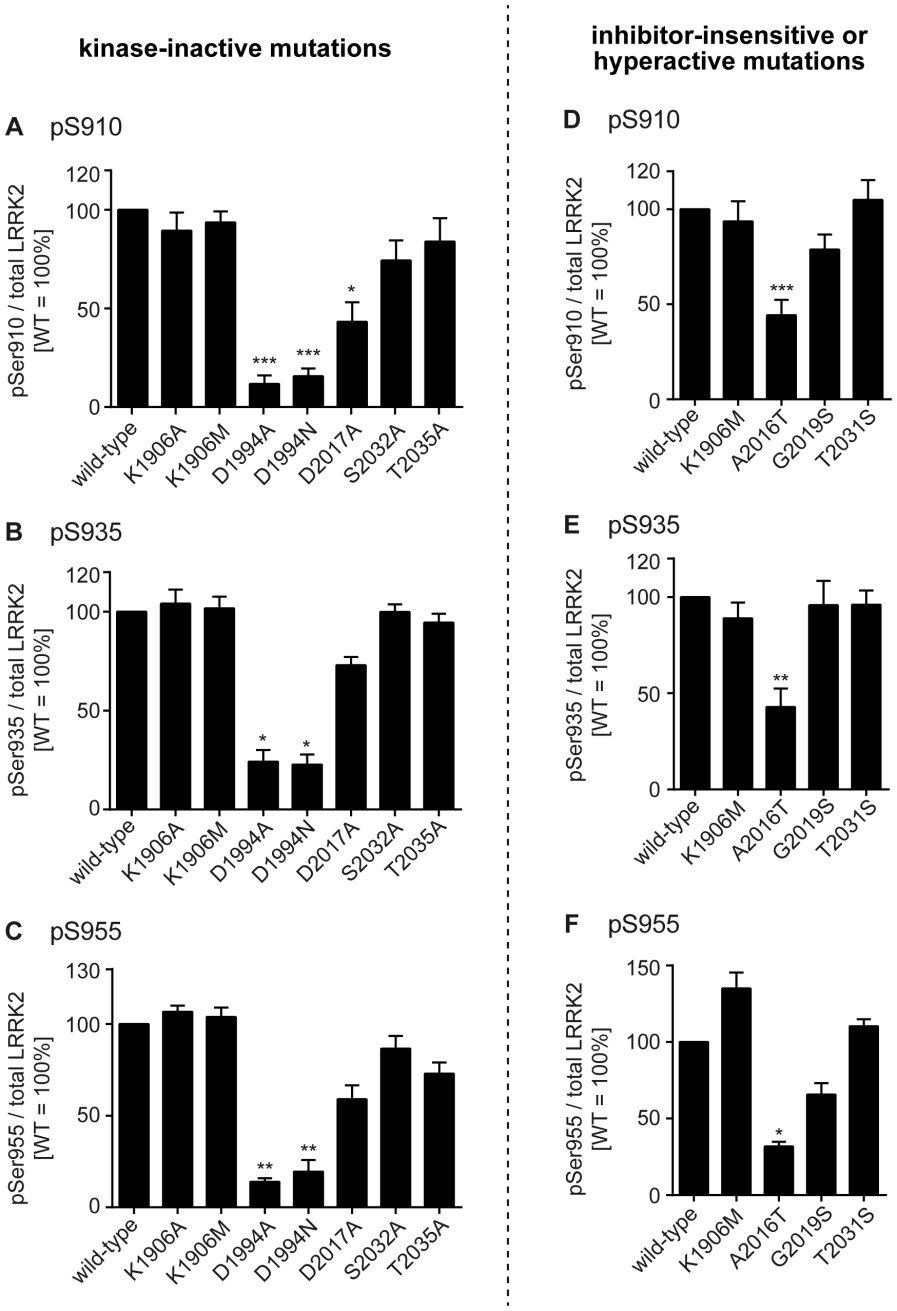
The basal phosphorylation of LRRK2 harboring kinase-modifying mutations. The combined results of quantification of the levels of basal phosphorylation at Ser910 (A and D), Ser935 (B and E), or Ser955 (C and F) of LRRK2 harboring the kinase-inactive mutations (A–C) and the inhibitor-insensitive or hyperactive mutations (D–F). The corresponding immunoblots are shown in Figure 3 and 4. The data are given as the percentage of those observed in WT LRRK2 (n = 6, mean ± standard error). *p<0.05, **p<0.01, and ***p<0.001 (Kruskal-Wallis test; comparison with WT LRRK2).

**Figure 3:**
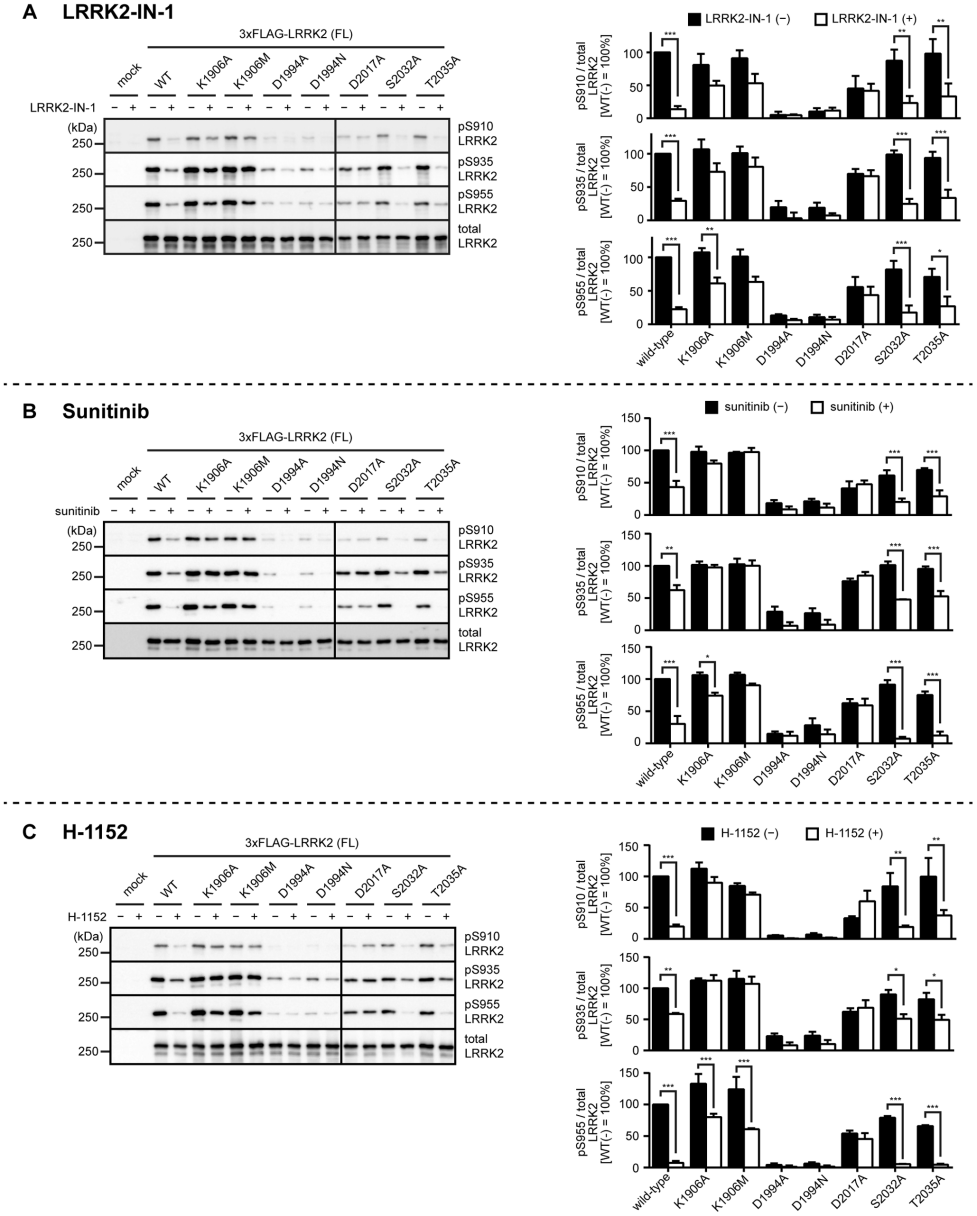
Inhibitor-induced dephosphorylation of kinase-inactive LRRK2. HEK293 cells transfected with wild-type, K1906A, K1906M, D1994A, D1994N, D2017A, S2032A or T2035A LRRK2 were treated with (A) 3 µM LRRK2-IN-1 or the solvent (0.1% DMSO) for 30 min, (B) 5 µM sunitinib or the solvent (0.1% DMSO) for 90 min, or (C) 30 µM H-1152 or the solvent (1% sterilized distilled water) for 90 min, and the phosphorylation of LRRK2 at Ser910, Ser935, or Ser955 was examined by immunoblotting. The levels of the phosphorylation were quantified and normalized by the expression levels of LRRK2 determined by immunoblotting with the anti-LRRK2 antibody (bottom panel). The data are given as the percentage of those observed in solvent-treated WT LRRK2 (n = 3, mean ± standard error). *p<0.05, **p<0.01, and ***p<0.001 (Two-way ANOVA test followed by Bonferroni’s test).

**Figure 4:**
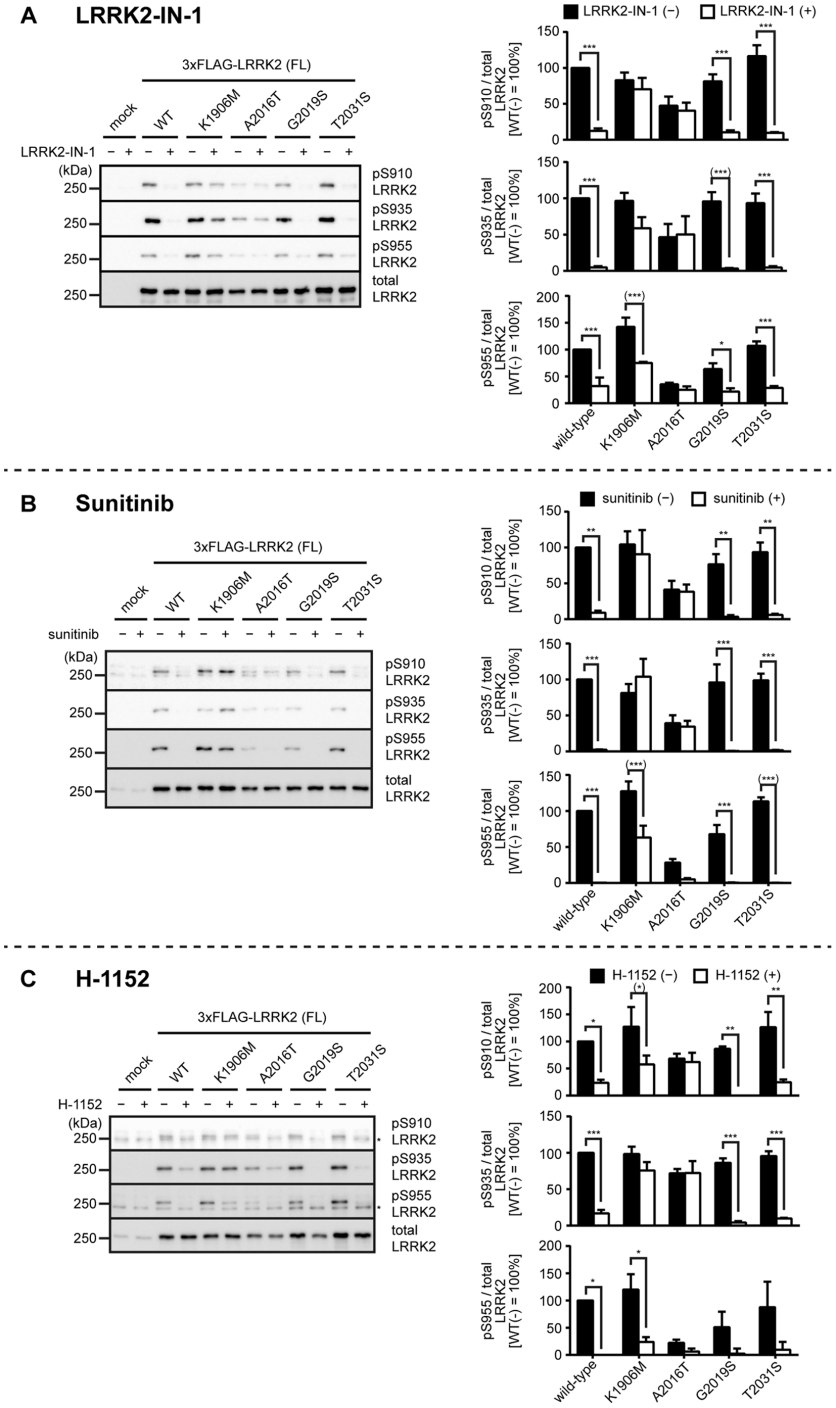
Inhibitor-induced dephosphorylation of inhibitor-resistant or hyperactive LRRK2. HEK293 cells transfected with wild-type, K1906M, A2016T, G2019S, T2031S LRRK2 were treated with (A) 3 µM LRRK2-IN-1 or the solvent (DMSO) for 30 min, (B) 5 µM sunitinib or the solvent (0.1% DMSO) for 90 min, or (C) 30 µM H-1152 or the solvent (1% sterilized distilled water) for 90 min, and the phosphorylation of LRRK2 at Ser910, Ser935, or Ser955 was examined by immunoblotting. Non-specific bands were marked with asterisks (*). The levels of the phosphorylation were quantified and normalized by the expression levels of LRRK2 determined by immunoblotting with the anti-LRRK2 antibody (bottom panel). The data are given as the percentage of those observed in DMSO-treated WT LRRK2 (n = 3, mean ± standard error). *p<0.05, **p<0.01, and ***p<0.001 (Two-way ANOVA followed by Bonferroni’s test). Asterisks with parentheses mean that the distribution of either sample did not follow a normal distribution (Shapiro-Wilk test).

We first confirmed the inhibitor-induced dephosphorylation of wild-type (WT) LRRK2 by overexpressing amino-terminally 3×FLAG-tagged full-length (FL) LRRK2 into HEK293 cells and treating them with LRRK2-IN-1, sunitinib, or H-1152 [15], [19]. We examined the phosphorylation of LRRK2 at three representative sites (Ser910, Ser935, and Ser955) by immunoblotting using their respective phosphorylation-specific antibodies. As reported previously, phosphorylation of WT LRRK2 at Ser910, Ser935, and Ser955 were detected (wild-type in Fig. 2–4), and these phosphorylations were significantly decreased upon treatment of cells with 3 µM LRRK2-IN-1 for 30 min, 5 µM sunitinib for 90 min, or 30 µM H-1152 for 90 min [15], [19] (wild-type in Fig. 3).

### Basal Phosphorylation of Kinase-inactive LRRK2

To investigate whether inhibition of the kinase activity of LRRK2 is involved in the inhibitor-induced dephosphorylation of LRRK2, we systematically characterized the inhibitor-induced dephosphorylation of LRRK2 mutants harboring kinase-inactive mutations. We utilized LRRK2 harboring K1906A, K1906M, D1994A, D1994N, D2017A, S2032A, and T2035A mutations as potentially kinase-inactive mutants, which have already been described in literature [34], [35]. When we compared the basal phosphorylation of these mutants at Ser910, Ser935, and Ser955, we found that the levels of phosphorylation were considerably variable among mutants: LRRK2 harboring K1906A, K1906M, S2032A, or T2035A mutations was phosphorylated at these sites to a similar extent to those in WT LRRK2, whereas LRRK2 harboring D1994A, D1994N, or D2017A mutation was phosphorylated at dramatically reduced levels compared with WT LRRK2 (Fig. 2A–C; Table 1: basal phosphorylation). According to the hypothesis depicted in Figure 1B, kinase-inactive LRRK2 neither activates the downstream kinase phosphorylating LRRK2 nor inhibits the downstream phosphatase dephosphorylating LRRK2. Theoretically, therefore, kinase-inactive LRRK2 should not be phosphorylated in the hot spot, which was not the case in our experiments (Fig. 1B, 2; Table 1: basal phosphorylation).

**Table 1:** Summary of the basal phosphorylation and inhibitor-induced dephosphorylation of kinase-inactive LRRK2.

	basal phosphorylation			inhibitor-induced dephosphorylation								
				LRRK2-IN-1			sunitinib			H-1152		
phospho-Ser	910	935	955	910	935	955	910	935	955	910	935	955
**wild-type**	+++	+++	+++	+++	++	+++	++	+	++	+++	+	+++
***theoretical kinase-inactive***	−	−	−	−	−	−	−	−	−	−	−	−
**K1906A**	+++	+++	+++	+	+	+	−	−	+	−	−	+
**K1906M**	+++	+++	+++	+	−	+	−	−	−	−	−	++
**D1994A**	+	+	+	−	+++	++	++	+++	−	+++	++	++
**D1994N**	+	+	+	−	++	+	+	++	+	+++	++	++
**D2017A**	+	++	++	−	−	−	−	−	−	−	−	−
S2032A*	++	+++	+++	++	+++	+++	++	++	+++	+++	+	+++
**T2035A**	+++	+++	++	++	++	++	++	+	+++	++	+	+++

**Figure 5:**
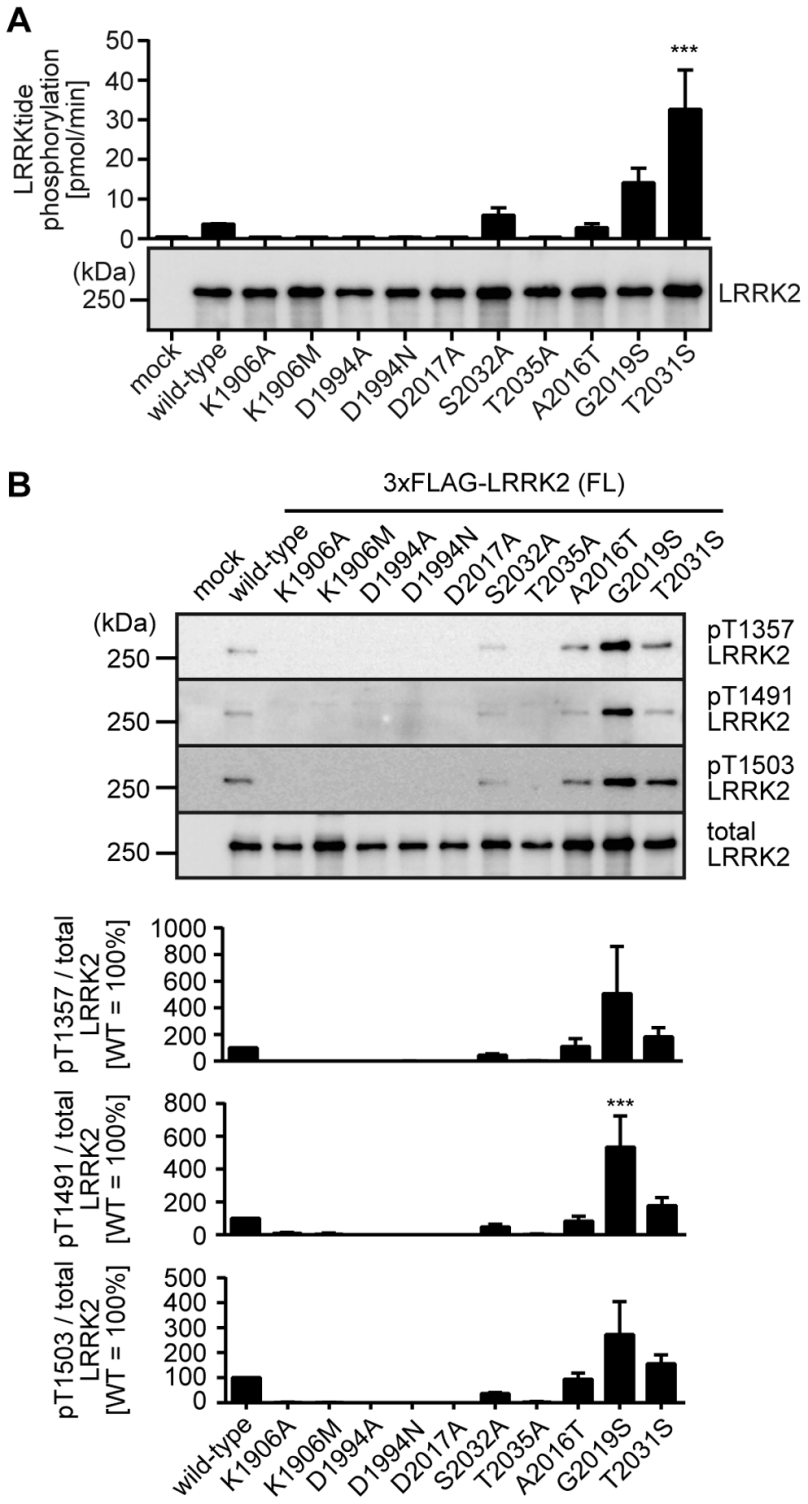
The kinase activity of LRRK2 harboring kinase-modifying mutations. (A) Phosphorylation of biotin-LRRKtide was examined by ELISA using an antibody that specifically recognizes phosphorylated LRRKtide. The data are given as the amount of phosphorylation per minute (n = 3, mean ± standard error). The amount of immunoprecipitated LRRK2 was examined by immunoblotting with an anti-LRRK2 antibody (bottom panel). (B) Autophosphorylations of WT and mutant LRRK2 at Thr1357, Thr1491, and Thr1503 were examined by immunoblotting with an antibody specifically recognizing corresponding phosphorylated threonines. The levels of the autophosphorylation were quantified and normalized by the expression levels of LRRK2 determined by immunoblotting with the anti-LRRK2 antibody (bottom panel). The data are given as the percentage of those observed in WT LRRK2 (n = 3, mean ± standard error). ***p<0.001 (One-way ANOVA followed by Bonferroni’s test).

Summary of the basal phosphorylation (Fig. 2A–C) and inhibitor-induced dephosphorylation of Ser910, Ser935, and Ser955 (Figure 3). Theoretical results of the kinase-inactive mutant according to the hypothesis schematized in the Figure 1B are also included (*theoretical kinase-inactive*). For basal phosphorylation, mutants showing 10∼50% phosphorylation at corresponding residues were marked with single plus sign (+). Likewise, those showing 50∼75% and 75∼100% phosphorylation were marked with double plus signs (++) and triple plus signs (+++), respectively. For inhibitor-induced dephosphorylation, mutants which remained 75∼100% phosphorylated after inhibitor treatment were marked with minus sign (−). Likewise, those remaining 50∼75%, 25∼50% and 0∼25% phosphorylated were marked with single plus sign (+), double plus signs (++) and triple plus signs (+++), respectively. *Note that S2032A LRRK2 retains the kinase activity based on our results (Figure 5).

### Inhibitor-induced Dephosphorylation of Kinase-inactive LRRK2

We next examined the inhibitor-induced dephosphorylation of LRRK2 harboring the kinase-inactive mutations using three LRRK2 inhibitors. Assuming that the hypothesis depicted in Figure 1B is the case, the kinase-inactive mutants should not undergo dephosphorylation upon treatment with inhibitors. However, we observed massive dephosphorylation of Ser910, Ser935, and Ser955 in T2035A LRRK2 upon treatment with the inhibitors (Fig. 3; Table 1: inhibitor-induced dephosphorylation). We also observed some dephosphorylation in K1906A, K1906M, D1994A, and D1994N LRRK2 (Fig. 3; Table 1). In contrast, D2017A LRRK2 failed to undergo dephosphorylation (Fig. 3; Table 1). Collectively, the response to inhibitors also significantly varied among kinase-inactive mutants, and notably, the extent of dephosphorylation in T2035A LRRK2 was similar to that of WT LRRK2. These results suggest that the inhibitor-induced dephosphorylation of LRRK2 does not require its kinase activity. One possible argument here is that the inhibitor-induced dephosphorylation of LRRK2 is due to an ‘off-target’ effect of inhibitors rather than inhibiting LRRK2 itself. LRRK2-IN-1 has been developed as a specific inhibitor of LRRK2, but a recent report has suggested that LRRK2-IN-1 has significant off-target effects in cells [36]. Sunitinib and H-1152 were first developed as inhibitors against receptor-type tyrosine kinases and Rho kinase, respectively, and therefore also are not specific to LRRK2 [37], [38]. Given the nonspecific nature of these inhibitors, it is still possible that a kinase responsible for the phosphorylation of LRRK2 at Ser910, Ser935 and Ser955 is directly inhibited by these inhibitors. This possibility should be investigated using a larger set of kinase inhibitors in the future. It has been reported that A2016T LRRK2 lacking Ala2016 essential for binding of inhibitors is resistant to LRRK2-IN-1, sunitinib, and H-1152 whereas it retains the normal kinase activity [15], [19]. This was confirmed in our experiments (Fig. 4 and 5; Table 2), ensuring that our experimental system is compatible with those used in previous studies. Moreover, the fact that A2016T LRRK2 lacking the ability to bind inhibitors fails to undergo inhibitor-induced dephosphorylation of Ser910 and Ser935 excludes the possibility that off-target pathways of inhibitors are responsible for the inhibitor-induced dephosphorylation of Ser910 and Ser935 of LRRK2. Interestingly, A2016T LRRK2 underwent apparent dephosphorylation of Ser955 upon treatment with inhibitors (Fig. 4; Table 2). Based on this observation, it is tempting to speculate that the kinase responsible for the phosphorylation of Ser955 might be sensitive to the inhibitors used in this study and distinct from that for Ser910 and Ser935.

**Table 2:** Summary of the basal phosphorylation and inhibitor-induced dephosphorylation of inhibitor-resistant or hyperactive LRRK2.

	basal phosphorylation			inhibitor-induced dephosphorylation								
				LRRK2-IN-1			sunitinib			H-1152		
phospho-Ser	910	935	955	910	935	955	910	935	955	910	935	955
**WT**	+++	+++	+++	+++	+++	++	+++	+++	+++	+++	+++	+++
**A2016T**	+	+	+	−	−	+	−	−	+++	−	−	++
**G2019S**	+++	+++	++	+++	+++	++	+++	+++	+++	+++	+++	+++
**T2031S**	+++	+++	+++	+++	+++	++	+++	+++	+++	+++	+++	+++

Summary of the basal phosphorylation (Figure 2D–F) and inhibitor-induced dephosphorylation of Ser910, Ser935, and Ser955 (Figure 4). A2016T is an inhibitor-resistant mutant, whereas G2019S and T2031S are hyperactive mutants (Figure 5). Results were classified using plus (+,++,+++) and minus (−) signs as described in Table 1.

### Basal Phosphorylation and Inhibitor-induced Dephosphorylation of Hyperactive LRRK2

Given that the kinase activity of LRRK2 is involved in the regulation of phosphorylation of LRRK2 in cells, hyperactive mutations should also affect the phosphorylation of LRRK2. To address this question, we examined the phosphorylation of LRRK2 at Ser910, Ser935, and Ser955 harboring G2019S or T2031S mutation which has been reported to upregulate the kinase activity [16], [39]. However, no difference in the levels of basal phosphorylation as well as the extent of inhibitor-induced dephosphorylation was observed (Fig. 2 and 4; Table 2). These results also suggested that the phosphorylation of LRRK2 in cells is not regulated by the kinase activity of LRRK2.

### Kinase Activity of Kinase-inactive, Inhibitor-resistant, and Hyperactive LRRK2

There might be a reasonable objection that the kinase-inactive mutants of LRRK2 examined in this study may maintain a certain amount of kinase activity, which results in their unexpected behavior in terms of the basal phosphorylation as well as the inhibitor-induced dephosphorylation. To address this issue, we re-examined the kinase activity of LRRK2 using two different *in vitro* assays. Transfected and immunoprecipitated 3×FLAG-LRRK2 was incubated with LRRKtide in the presence of ATP for 15 min at 30°C, and phosphorylation of LRRKtide was detected by enzyme-linked immunosorbent assay (ELISA). We also examined the autophosphorylation of LRRK2 at Thr1357, Thr1491, and Thr1503 by immunoblotting using their respective phosphorylation-specific antibodies [31], [33] We confirmed that LRRK2 harboring K1906A, K1906M, D1994A, D1994N, D2017A, or T2035A mutation lacks both the kinase activity and autophosphorylation (Fig. 5). These results ensure that the kinase-inactive mutants used in this study except S2032A LRRK2 lack the kinase activity *in vitro*. We also confirmed that the hyperactive mutants exhibited increased levels of kinase activity compared with WT LRRK2 (Fig. 5). It is still possible that the kinase activity of LRRK2 examined in *in vitro* assays does not correlate with that within cells. However, for example, if K1906A/M and T2035A LRRK2 retain some kinase activity in cells and the residual kinase activity enables them to regulate the kinase/phosphatase responsible for phosphorylating Ser residues in the hot spot of LRRK2, there still remains another discrepancy that K1906M LRRK2 is less competent to be dephosphorylated in response to the inhibitors, whereas T2035A LRRK2 undergoes dephosphorylation (inhibitor-induced dephosphorylation in Fig. 3; Table 1).

The kinase-inactive mutations of LRRK2 examined in this study disrupt its kinase activity in different manners. For example, Lys1906 and Asp2017 which are equivalent to Lys72 and Asp184 in cAMP-dependent protein kinase (PKA) are supposed to be required for binding ATP (or ATP-competitive inhibitors) with proper orientation, whereas Asp1994 and Thr2035 which are equivalent to Asp166 and Thr201 in PKA are supposed to be required for positioning the catalytic loop and not directly involved in binding of ATP [40]. Considering that mutants deficient in GTP binding [30] as well as familial Parkinson mutants [16], [18] exhibit reduced phosphorylation in cells, it is tempting to speculate that binding of ATP-competitive inhibitors to LRRK2 would induce a dephosphorylation-prone conformation, which might lead to dephosphorylation of LRRK2. If this were the case, the extent of inhibitor-induced dephosphorylation would depend on the affinity or the binding mode of inhibitors to LRRK2. Indeed, this speculation agrees well with our results showing that K1906A/M and D2017A LRRK2 were less competent to undergo inhibitor-induced dephosphorylation compared with D1994A/N and T2035A LRRK2.

In summary, we identified a clear counterexample (T2035A) to the hypothesis that the phosphorylation of LRRK2 is regulated by the kinase activity of LRRK2, showing that inhibition of the kinase activity of LRRK2 is dispensable for the inhibitor-induced dephosphorylation of LRRK2 in cells. Further investigation into the conformational change of LRRK2 would elucidate the mechanism of the inhibitor-induced dephosphorylation of LRRK2 and that of the familial Parkinson mutations.
